# Improved reproducibility of simple quantitative indices from ^99m^Tc-GSA liver functional imaging

**DOI:** 10.1007/s12149-013-0689-5

**Published:** 2013-04-18

**Authors:** Gou Ogasawara, Yusuke Inoue, Yoshihiro Itoh, Satomi Tagami, Keiji Matsunaga, Kenji Miki

**Affiliations:** 1 Department of Diagnostic Radiology, Kitasato University School of Medicine, 1-15-1 Kitasato, Minami-ku, Sagamihara, Kanagawa 252-0374 Japan; 2 Department of Radiology, Kitasato University East Hopital, 2-1-1 Asamizodai, Minami-ku, Sagamihara, Kanagawa 252-0380 Japan; 3 Department of Surgery, Showa General Hospital, 8-1-1 Hanakoganei, Kodaira, Tokyo 187-8510 Japan

**Keywords:** ^99m^Tc-GSA, Quantitation, Region of interest, Reproducibility, Liver function

## Abstract

**Objective:**

We evaluated intra- and interoperator reproducibilities in calculating the conventional indices HH15 and LHL15 from ^99m^Tc-diethylenetriamine pentaacetic acid galactosyl human serum albumin (^99m^Tc-GSA) scintigraphy, and proposed new, simple methods for the calculation of quantitative indices.

**Methods:**

The results of ^99m^Tc-GSA scintigraphy in 33 patients were retrospectively analyzed. Heart and liver ROIs were drawn manually to cover cardiac blood pool and entire liver, respectively, and HH15 and LHL15 were calculated. In addition, square regions of interest (ROIs) of fixed sizes were placed at the highest activity in blood pool and the liver. Using the square heart ROI, sHH15, an equivalent of HH15, was computed. Fractional liver uptake at 15 min (FLU15) was calculated using the square heart and liver ROIs. Intra- and interoperator reproducibilities, as well as correlation with Indocyanine green retention rate at 15 min (ICG R15), were assessed for these four indices by linear regression analysis.

**Results:**

Substantial intra- and interoperator variabilities were found for HH15 and LHL15. The correlation coefficients for intra- and interoperator comparisons were 0.884 and 0.869 for HH15, respectively, and 0.919 and 0.917 for LHL15, respectively. The use of square ROIs instead of hand-drawn ROIs improved reproducibility. The correlation coefficients for intra- and interoperator comparisons were 0.988 and 0.973 for sHH15, respectively, and 0.989 and 0.975 for FLU15, respectively. Correlation with ICG R15 was better for sHH15 (*r* = 0.619) and FLU15 (*r* = −0.656) than for HH15 (*r* = 0.439) and LHL15 (*r* = −0.490).

**Conclusions:**

HH15 and LHL15 showed substantial intra- and interoperator variabilities, and the use of square ROIs are indicated to provide better reproducibility.

## Introduction

^99m^Tc-diethylenetriamine pentaacetic acid galactosyl human serum albumin (^99m^Tc-GSA) is recognized by asialoglycoprotein receptors expressed on the hepatocyte membrane specifically [1]. After intravenous injection, it is distributed in blood pool and is gradually taken up exclusively by the hepatocytes. The rates of blood clearance and hepatic uptake depend on the functional hepatocyte mass, and quantitative evaluation of the kinetics of ^99m^Tc-GSA permits the assessment of hepatic functional reserve. Single photon emission computed tomography with this agent demonstrates regional hepatic function, and contributes to preoperative evaluation in patients with liver tumors.

For quantitative evaluation of ^99m^Tc-GSA kinetics, methods to determine the amount of asialoglycoprotein receptors using compartment models have been proposed [2–5]. In clinical practice, however, simple quantitative indices, HH15 and LHL15, are widely used [6]. HH15 represents the rate of blood clearance and LHL15 represents the degree of hepatic accumulation. For the calculation of these indices, irregular-shaped regions of interest (ROIs) covering the cardiac blood pool and the liver are drawn manually by an operator on anterior dynamic images. HH15 and LHL15 are readily calculated from heart and liver counts, and have been indicated to reflect hepatic functional reserve [6–9].

HH15 and LHL15 vary depending on ROI setting, which may impair the reliability of these indices [10, 11]. Reproducible ROI setting appears to be difficult, especially for cardiac blood pool because of its ill-defined upper border. In addition, cross-contamination between cardiac blood pool and the liver is inevitable, because they neighbor each other. Scattering photons originating from the liver may be counted within the ROI for cardiac blood pool. Respiratory motion results in overlapping of cardiac blood pool and the liver. In the present study, we evaluated intra- and interoperator reproducibilities in calculating HH15 and LHL15 from ^99m^Tc-GSA scintigraphy, and proposed new, simple methods for the calculation of quantitative indices.

## Materials and methods

### Subjects

The results of ^99m^Tc-GSA scintigraphy performed for clinical indications in 33 patients (21 men and 12 women; 64.9 ± 10.8 years, mean ± SD) were retrospectively analyzed. Indocyanine green retention rate at 15 min (ICG R15) was measured within 2 months of ^99m^Tc-GSA scintigraphy in 30 patients (27 and 3 patients in Child-Pugh classes A and class B, respectively). The institutional review board approved the current retrospective study, and need for informed consent was waived.

### Imaging procedures

The patients were injected intravenously with 185 MBq of ^99m^Tc-GSA (3 mg) and underwent anterior dynamic imaging for 25 min. The field-of-view was set to cover the heart and liver. A gamma camera (e.cam+, Siemens, Erlangen, Germany) equipped with a low-energy high-resolution collimator was used. Data were acquired using a 15 % energy window centered at 140 keV. Matrix size was 128 × 128, zoom factor was 1.45, and pixel size was 3.3 mm.

### Data analysis

**Fig. 1:**
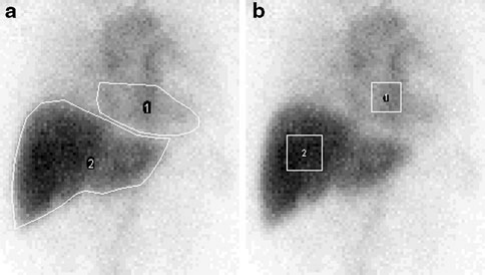
Hand-drawn (**a**) and square (**b**) ROIs set for cardiac blood pool and the heart

ROIs were set using ImageJ 1.46 software. An operator placed four types of ROIs for each image set. Heart and liver ROIs were drawn manually to cover cardiac blood pool and entire liver, respectively (Fig. 1a). In addition, a square ROI of 10 × 10 pixels was set at the highest activity in blood pool, and was defined as a square heart ROI (Fig. 1b). The square heart ROI mainly included cardiac blood pool but was allowed to include activity in the great vessels above cardiac blood pool. A square ROI of 12 × 12 pixels was set at the highest activity in the liver, and was defined as a square liver ROI. Hand-drawn and square heart ROIs were taken on the 2-min image, and hand-drawn and square liver ROIs were taken on the 15-min image. The positions of the square ROIs were determined visually by the operator to maximize the total counts in the ROIs.

An operator (operator A) performed ROI setting twice at an interval of 2 weeks or longer to assess intraoperator reproducibility. Another operator (operator B) performed ROI setting independently to assess interoperator reproducibility. Both operators were radiological technologists, and operators A and B had 11- and 6-year experiences in nuclear medicine practice, respectively.

### Functional indices

HH15 and LHL15 were calculated using the following equations:

1 $$ {\text{HH15 }} = {\text{ H15}}/{\text{H3}} $$

2 $$ {\text{LHL15 }} = {\text{ L15}}/\left( {{\text{H15 }} + {\text{ L15}}} \right) $$

where H3 and H15 are counts in the heart ROI at 3 and 15 min, respectively, and L15 is count in the liver ROI at 15 min. Total counts acquired during 2.5–3 and 14.5–15 min in each ROI were defined as counts at 3 and 15 min, respectively.

Using the square ROI, sHH15, an equivalent of HH15, was computed using the following equation:

3 $$ {\text{sHH15 }} = {\text{ sH15}}/{\text{sH3}} $$

where sH3 and sH15 are the counts in the square heart ROI at 3 and 15 min, respectively.

In addition, fractional liver uptake at 15 min (FLU15) was calculated from counts in the square ROIs. The ratio of the total radioactivity in blood pool to the counts in the square heart ROI and that of the total activity in the liver to the counts in the square liver ROI are assumed to be constant irrespective of time after injection, and are defined as *K*1 and *K*2, respectively. Assuming radioactivity after ^99m^Tc-GSA injection exists exclusively in blood pool and the liver, the following equations hold:

4 $$ K 1\times {\text{sL3 }} + \, K 2\times {\text{sH3 }} = \, D $$

5 $$ K 1\times {\text{sL15}} \times C \, + \, K 2\times {\text{sH15}} \times C \, = \, D $$

where *D* is injected radioactivity, *C* is a correction factor for physical decay of ^99m^Tc from 3 to 15 min, i.e., 1.0233, and sL3 and sL15 are the counts in the square liver ROI at 3 and 15 min, respectively. FLU15 is defined as follows:

6 $$ {\text{FLU15 }} = \, (K 1\times {\text{sL15}} \times C)/D $$

The combination of Eqs. (4)–(6) yields

7 $$ {\text{FLU}}15\, = \,{{{\text{sL}}15\, \times \,\left( {{\text{sH}}3 - {\text{sH15}}\, \times \,{\text{C}}} \right)} \mathord{\left/ {\vphantom {{{\text{sL}}15\, \times \,\left( {{\text{sH}}3 - {\text{sH15}}\, \times \,{\text{C}}} \right)} {\left( {{\text{sL15}}\, \times \,{\text{sH3}} - {\text{sL3}}\, \times \,{\text{sH15}}} \right)}}} \right. \kern-0pt} {\left( {{\text{sL15}}\, \times \,{\text{sH3}} - {\text{sL3}}\, \times \,{\text{sH15}}} \right)}} $$

for the calculation of FLU15 from measured counts.

Linear regression analysis was performed by the least-square method for the functional indices (HH15, LHL15, sHH15, and FLU15) obtained by operator A’s two measurements (intraoperator reproducibility) and for those obtained by operators A and B (interoperator reproducibility). Linear regression analysis was also performed between functional indices and ICG R15 in 30 patients in whom ICG R15 was available. Functional indices from the first measurement by operator A were used for this analysis. Similarly, HH15 and FLU15 were compared with the sHH15.

## Results

**Fig. 2:**
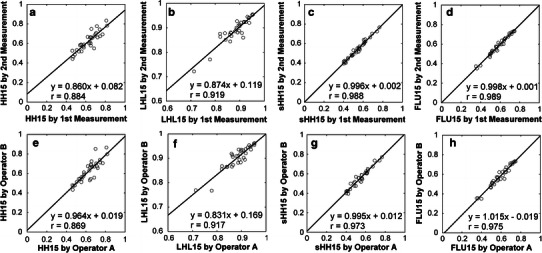
Results of intra- and interoperator reproducibilities. The* upper row* shows interoperator reproducibility of HH15 (**a**), LHL15 (**b**), sHH15 (**c**), and FLU15 (**d**), and the *lower row* shows the intraoperator reproducibility of HH15 (**e**), LHL15 (**f**), sHH15 (**g**), and FLU15 (**h**). The *lines* represent the regression lines

**Fig. 3:**
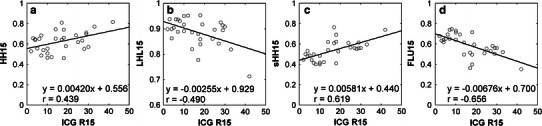
Relationship of HH15 (**a**), LHL15 (**b**), sHH15 (**c**), and FLU15 (**d**) with ICG R15. The *lines* represent the regression lines

**Fig. 4:**
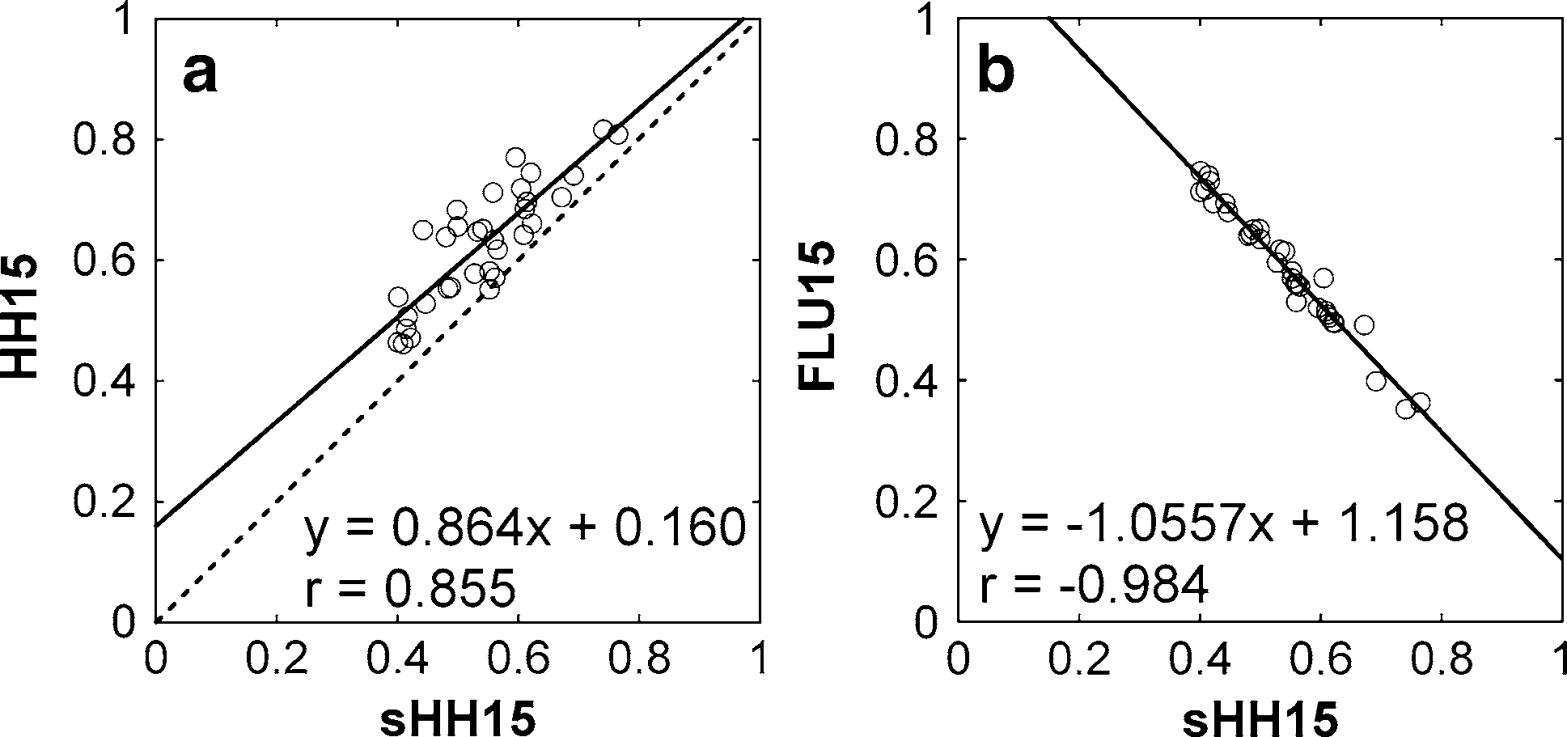
HH15 (**a**) and FLU15 (**b**) plotted against sHH15. The *solid line* represents the regression line. The *broken line* in panel **a** represents the line of identity, respectively

Substantial intra- and interoperator variabilities were found for HH15 and LHL15 (Fig. 2). The correlation coefficients for intra- and interoperator comparisons were 0.884 and 0.869 for HH15, respectively, and 0.919 and 0.917 for LHL15, respectively. The use of square ROIs instead of hand-drawn ROIs improved reproducibility. The correlation coefficients for intra- and interoperator comparisons were 0.988 and 0.973 for sHH15, respectively, and 0.989 and 0.975 for FLU15, respectively. Correlation with ICG R15 was better for sHH15 (*r* = 0.619) and FLU15 (*r* = −0.656) than for HH15 (*r* = 0.439) and LHL15 (*r* = −0.490) (Fig. 3). HH15 was correlated well with sHH15 (*r* = 0.855) but was systematically larger than sHH15 (Fig. 4a). Correlation between sHH15 and FLU15 was excellent with a correlation coefficient of −0.984 (Fig. 4b).

## Discussion

In this study, HH15 and LHL15 were correlated with ICG R15; however, they showed substantial intra- and interoperator variabilities. The use of square ROIs of fixed sizes, instead of hand-drawn ROIs, provided functional indices with better reproducibility and higher correlation with ICG R15. An operator needs only to move the predetermined ROIs to include the highest activity in blood pool and the liver. The modified indices sHH15 and FLU15 may reflect hepatic functional reserve better than HH15 and LHL15 with less operator’s burden and appear to be suitable for clinical use. The clinical utility of these modified indices is indicated to be worth investigating in the future.

Both HH15 and sHH15 are calculated by dividing blood pool activity at 15 min by that at 3 min and represent the rate of blood clearance of ^99m^Tc-GSA. They should be identical ideally; however, HH15 was larger than sHH15 actually, which appears to be because the heart counts estimated using a hand-drawn ROI covering the cardiac blood pool were contaminated by hepatic activity especially at 15 min. The use of a square ROI placed at the highest activity in blood pool is indicated to have reduced such contamination, presumably resulting in better accuracy in assessing blood clearance. In the present study, a 15 % energy window was set for image acquisition. A 20 % window, another common option, contains more scatter fraction. The use of a 20 % window may increase the effect of hepatic activity on heart counts and, consequently, overestimation of HH15, which is the subject of a future study.

In clinical practice, LHL15 reflecting hepatic accumulation is used in addition to HH15 reflecting blood clearance. We calculated the FLU15 as an alternative index of hepatic accumulation using square ROIs. FLU15 was shown to be highly reproducible and to be correlated with ICG R15, similarly with sHH15. It may serve as a useful indicator of hepatic functional reserve. In the calculation of FLU15, injected radioactivity was distributed exclusively in blood pool and the liver. ^99m^Tc-GSA shows high in vivo stability and is taken up by the liver and not by any other organ. Radioactive materials are extracted through the biliary and urinary systems slowly [7]. The assumption for the calculation of FLU15 appears to be valid during an early period after injection. FLU15 was closely correlated with sHH15, indicating that both indices represent the kinetics of ^99m^Tc-GSA faithfully. The use of only one of the parameters may suffice because of the close correlation. Increase in hepatic functional reserve results in increase in FLU15 and decrease in HH15. Positive, although not proportional, relationship between functional reserve and FLU15 may help understanding. However, measurement of the amount of asialoglycoprotein receptors using a compartment model would be desirable, because it provides an index of hepatic functional reserve that is proportional to functional hepatocyte mass [12].

Correlations of HH15 and LHL15 with ICG R15 were relatively low in comparison with the previous studies [6–11]. Most subjects belonged to Child-Pugh classes A in the present study, and the low correlations may be attributable to the small variations in hepatic functional reserve. Because the patients studied tended to have preserved liver function, further studies are required in patients with severe liver damage. The validity of the calculation of FLU15 should be also tested in patients with heart failure, because prolonged circulation and increased peripheral blood pool may affect the calculation.

In summary, we investigated the methods for simple, quantitative assessment of hepatic functional reserve in ^99m^Tc-GSA scintigraphy and demonstrated that the use of square ROIs improves reproducibility. Indices obtained using square ROIs are indicated to be superior to the conventional indices HH15 and LHL15.
